# Developmental Trajectories of Intrinsic Capacity Among Older Adults: Results from the China Longitudinal Study of Aging

**DOI:** 10.3390/healthcare13050520

**Published:** 2025-02-27

**Authors:** Jia Zhou, Hui Chang, Zhiwen Wang

**Affiliations:** 1 School of Nursing, Peking University, Beijing 100191, China; 2111110233@bjmu.edu.cn; 2 School of Nursing, Guizhou Medical University, Guiyang 561113, China; cc120508@126.com

**Keywords:** functional status, influencing factors, cohort studies, healthy aging

## Abstract

**Background**: Previous studies have shown that intrinsic capacity changes over time and can independently predict adverse outcomes such as mortality and care dependence in older adults. However, explorations of the heterogeneity in the developmental trajectories of intrinsic capacity are limited. **Aim:** This study aimed to identify potential intrinsic capacity trajectory groups and the factors impacting different trajectory groups among older adults. **Methods**: We utilized data from 2454 older adults aged 60 and above, sourced from the Chinese Longitudinal Healthy Longevity Survey. Our analyses were conducted using growth mixture modeling, chi-square tests, and multinomial logistic regression analysis. **Results**: We identified four intrinsic capacity trajectory groups among older adults in China: low-level intrinsic capacity (3.2%), medium-level increasing intrinsic capacity (13.0%), medium-level decreasing intrinsic capacity (12.0%), and stable high intrinsic capacity (71.8%). Age was an influencing factor of the medium-level increasing intrinsic capacity, medium-level decreasing intrinsic capacity, and stable high intrinsic capacity trajectory groups. Compared to individuals in the low-level intrinsic capacity trajectory group, individuals in the medium-level decreasing intrinsic capacity group were more likely to regularly exercise and participate in social activity, and those in the stable high intrinsic capacity group were more likely to be male, drink, participate in social activity, and have good self-rated health. **Conclusions**: Understanding the developmental trajectories of the intrinsic capacity of the older adults can contribute to formulating personalized intervention planning. We identified four intrinsic capacity trajectories in a cohort of older adults in China, which highlights significant heterogeneity in intrinsic capacity development. Our findings suggest that age, gender, exercise, drinking, social activity, and self-rated health of older adults have important effects on different intrinsic capacity development trajectories.

## 1. Introduction

Population aging has emerged as a prevalent trend in global demographic development. A survey shows that the global older adult population is expected to increase to 2.1 billion by 2050 [1]. In China, the trend of population aging is particularly severe due to its large population base, the increasing life expectancy of older adults, and the low fertility rate [2]. According to the latest census data from 2021, the number of older adults aged 60 and above in China has reached 249 million, comprising 17.9% of the country’s total population [3]. As the population ages, the healthcare needs of older adults increase. How to develop and maintain the health-related functions of older adults to facilitate healthy aging has become an urgent global issue to be addressed [4]. The functional performance of an individual is determined by its intrinsic capacity (IC), the environment, and the interaction between the two. Therefore, the level of IC is an important indicator of health outcomes in older adults, and understanding the level and trends of IC development is a key aspect of promoting healthy aging in older adults [5].

The concept of IC was first introduced in the *World Report on Ageing and Health* published by the World Health Organization (WHO) in 2015 [6], which identified IC as the combination of all physical and mental abilities that an individual may draw upon as they age. IC mainly includes five key domains: cognition, locomotion, sensory (including vision and hearing), vitality, and psychological [7]. To enable better application of IC, the integrated care for older people (ICOPE) screening tool was further proposed in a WHO handbook about guidelines on person-centered assessments and pathways in primary care in 2019, and the use of the ICOPE screening tool for the initial screening of older adults for IC was recommended [7,8]. The handbook covers six priority conditions of IC, namely, cognitive decline, limited mobility, malnutrition, visual impairment, hearing loss, and depressive symptoms. This tool provides an important reference for further in-depth assessment of the IC of older adults and targeted implementation of interventions. Aging is a dynamic developmental process [9]. Therefore, IC is a dynamic indicator that changes over time [3,10,11]. The WHO also showed in its world report on aging and health that in the process of aging, an individual’s level of IC will generally go through three stages: high and stable, decreasing, and significantly decreasing [6]. In addition, the IC of older adults will also decline before they experience adverse life events [6]. It can be seen that there is heterogeneity in the changes in the IC of older adults. Different developmental trajectories of IC may imply different causes of symptoms and have different effects on patient outcomes. Therefore, clarifying the developmental trajectories of IC in Chinese older adults is of great practical importance to improve the health outcomes of the older adults and thus promote their healthy aging. In recent years, while the number of studies on the IC of older adults has grown, most research still only describes a specific point in time, without long-term tracking or comparative analysis of changes in IC across different groups. Additionally, current research falls short in sample selection, data collection, and analytical methods, making it difficult to fully capture the complexity and dynamics of older adults’ IC.

The IC of older adults is influenced by a variety of factors, and by exploring the influencing factors of different trajectories of change, we can provide more precise interventions to enhance the IC of older adults. Relevant studies have shown that sociodemographic information (e.g., age, gender, literacy, economic level), lifestyle (diet, exercise), disease factors (dementia, coronary heart disease, COPD, osteoarthritis), social environment, and social security system are all influential factors in the IC of older adults [5,12,13,14,15,16]. Although previous studies have explored the factors influencing the IC of older adults in terms of individuals, social backgrounds, lifestyles, and social environments, most of the existing studies are cross-sectional studies, which can only understand the status of the IC at a certain stage or period but cannot dynamically monitor the trend of changes in the IC of older adults [17]. However, longitudinal studies can identify the trajectory of changes in the IC of older adults through tracking over a period of time, which is helpful to identify the time points when the IC of older adults needs intervention to achieve precise intervention.

Continuous monitoring is essential to understand the trajectory of changes in the IC of older adults, but longitudinal studies on the IC of older adults in China are not sufficient [18]. Therefore, this study will use nationally representative data to explore the heterogeneous trajectories of IC among older adults in China and the characteristics of the different trajectories. In addition, this study will explore the factors influencing the different trajectories of IC to provide a basis for promoting an understanding of the process of change in the IC of older adults and developing interventions to slow down the decline in their IC levels.

## 2. Materials and Methods

### 2.1. Study Design and Participants

This longitudinal study was conducted to identify the groups of IC developmental trajectory through a secondary analysis and to analyze the factors affecting each group. The data we used were from the Chinese Longitudinal Healthy Longevity Survey (CLHLS), a collaborative effort between Duke University in the United States and Peking University in China. The CLHLS conducted face-to-face interviews with individuals in 1998, with a subsequent follow-up and recruitment of new participants in 2000, 2002, 2005, 2008, 2011, 2014, and 2018 using internationally compatible questionnaires [19]. All participants provided informed consent forms [19,20].

**Figure 1:**
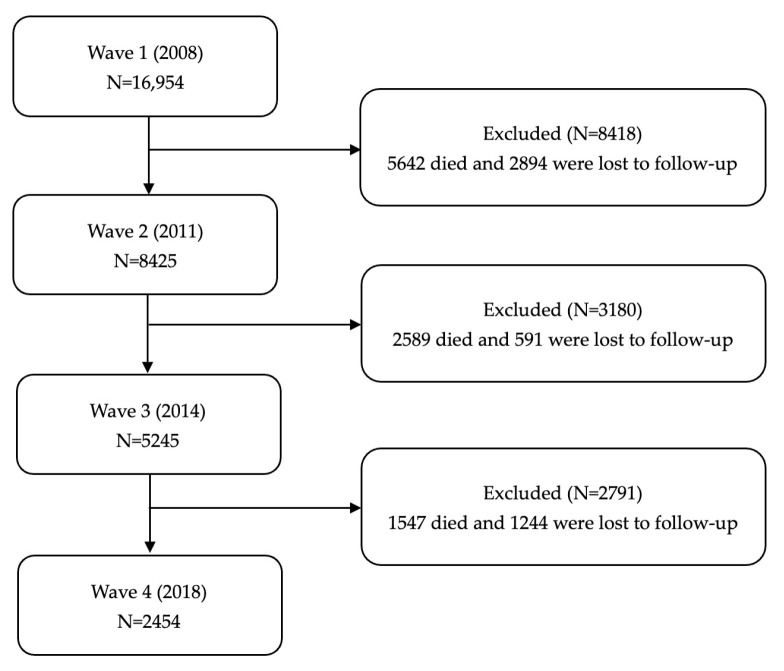
The flowchart of the analytic sample selection process.

For the current study, we used data from the last four waves of datasets, including 2008, 2011, 2014, and 2018. Publicly available databases were downloaded from the CLHLS webpage (https://agingcenter.duke.edu/CLHLS, accessed on 1 March 2024). The analysis sample was restricted to older adults aged ≥ 60 years old at baseline in 2008 (n = 16,954). In addition, participants were excluded if they died, were lost to follow-up, or failed to report the assessment result of IC. Ultimately, a total of 2454 of the 2008 initial interviewees who were re-interviewed in 2018 were included. The flowchart of the analytic sample selection process is shown in Figure 1.

### 2.2. Measures

IC was set as a dependent variable. Sociodemographic characteristics (age, gender, marital status, co-residence with household member(s), financial support, self-rated economic status), lifestyles (smoking, drinking, exercise, social activity), social services (medical service, retirement pension), life satisfaction, and self-rated health were set as independent variables.

#### 2.2.1. IC

According to the WHO ICOPE guideline [7], IC includes five domains: locomotion, vitality, sensory (hearing and vision), cognition, and psychological capacity. The score for each domain was dichotomized as 1 = “impaired” and 0 = “not impaired”. The total IC composite score summed the number of each normal domain and ranged from 0 to 6 in this study. Each domain of IC decline was recorded as 1 point; 0 points indicated no IC decline, and the higher the score was, the greater the IC decline [8].

The locomotion domain was measured through three tests (performed by trained technicians) [21]. The tests included picking up a book from the floor, standing up from a chair, and turning approximately 360°. Thus, three dichotomous indicators were used to represent locomotion impairment. If participants were not able to perform any of the tests independently, they were considered to have locomotion impairment.

The vitality domain was assessed by body mass index (BMI), which is similar to previous studies in IC [12,22]. The anthropometric measurements were carried out to obtain height (in meters) and weight (in kilograms) by the interview in the CLHLS. BMI was calculated as weight divided by the square of height. BMI was categorized as obese (≥28 kg/m^2^), overweight (24–27.9 kg/m^2^), normal (18.5–23.9 kg/m^2^), and underweight (<18.5 kg/m^2^) [23]. If the BMI of an individual was obese, overweight, or underweight, it was considered an impairment in vitality.

The sensory domain included vision and hearing function assessments [12,22]. Vision impairment was assessed by the question, “Can the interviewee see a break in the circle on the cardboard sheet when lit by a flashlight and distinguish where the break is located?” Hearing impairment was assessed by the question, “Do you have any difficulty with your hearing?”

The cognition domain was evaluated by the Chinese version of the Mini-Mental State Examination (MMSE) [24,25]. The MMSE consists of 24 items, including general ability (3 items), responsiveness (3 items), attention and calculation ability (6 items), recall (3 items), and language comprehension and self-coordination (6 items). Only one item, “number of kinds of food named in 1 min”, scored 0–7, and the other items were scored on a dichotomous scale (1 = correct, 0 = wrong), so the score range of the cognition domain was 0–30. The cut-off value was set at ≤24, indicating an impairment in cognition.

The psychological domain was assessed by two sets of questions in 2008 (Do you often feel fearful or anxious? Do you often feel lonely and isolated? Do you feel the older you get, the more useless you are?), 2011, and 2014 (Have you felt sad, blue, or depressed for two weeks or more in the last 12 months? Have you lost interest in most things, like hobbies, work, or similar activities?). The 10-item Center for Epidemiologic Studies Depression Scale (CES-D-10) was used to assess depressive symptoms in 2018 [26]. The CES-D-10 includes ten items using a four-point metric, “rarely or never/seldom”, “sometimes”, “often”, and “always”, which were assigned scores of 0, 1, 2, and 3 points, respectively. The total score ranged from 0 to 30 points. A higher score indicated greater severity of depressive symptoms. A score ≥ 10 on the CES-D-10 was considered to indicate depression, as validated by previous studies.

#### 2.2.2. Independent Variables

The following demographic characteristics were included: age, gender, marital status, co-residence with household member(s), financial support, and self-rated economic status (age: 0 = ≥80, 1 = 60~69, 2 = 70~79; gender: 0 = female, 1 = male; marital status: 0 = never married, 1 = married, 2 = divorced, 3 = widowed; co-residence with household member(s): 0 = no, 1 = yes; financial support: 0 = no, 1 = yes; self-rated economic status: 0 = poor, 1 = not bad, 2 = rich).

Lifestyles: smoking, drinking, exercise, and social activity (Do you smoke at present? 0 = no, 1 = yes; Do you drink alcohol at present? 0 = no, 1 = yes; Do you exercise regularly at present? 0 = no, 1 = yes; Do you now perform social activities regularly? 0 = no, 1 = yes) assessed at baseline were included.

Social services: medical service, retirement pension (Can you get adequate medical service when you are sick? 0 = no, 1 = yes; Do you have a retirement pension at present? 0 = no, 1 = yes) assessed at baseline were included.

Life satisfaction and self-rated health (How do you rate your life at present? 0 = bad, 1 = good; How do you rate your health at present? 0 = bad, 1 = good) assessed at baseline were included.

### 2.3. Statistical Analysis

Data analysis was performed by SPSS statistics 27.0 and Mplus 8.3. The descriptive statistical analysis of participants’ general characteristics was carried out by SPSS statistics 27.0. Moreover, the growth mixture modeling (GMM) was used to explore the different trajectory groups of IC of older adults. To select the best number of potential categories, first set a single category growth model and then gradually increase the number of categories to compare the fitting indicators between models, combined with the actual significance and statistical indicators to determine the best model. Model evaluation included Akaike Information Criteria (AIC), Bayesian Information Criteria (BIC), Sample-Size-Adjusted BIC (SABIC), entropy index, Lo–Mendel–Rubin Likelihod Ratio Test (LMR-LRT), and Bootstrapped Likelihood Ratio Test (B-LRT) [27]. The AIC, BIC, and SABIC are indices based on the number of sub-trajectories, sample size, or estimated parameters to compare the explanatory power and simplicity of the model, whereby a smaller value indicates better model fitting [28]. The entropy index was used to check whether individual cases were accurately grouped into the correct group. Its values range from 0 to 1; the closer to 1, the more accurate the classification [29]. The VLMR-LRT and BLRT are used to compare the results of the k class model and the K-1 class model. The significant *p*-value indicates that the k class model is better than the k-1 class model [30]. Finally, to determine the influencing factors of each trajectory group in the IC of older adults, the classified trajectory groups were taken as the dependent variables, and the sociodemographic characteristics, lifestyles, social services, life satisfaction, and self-rated health of older adults were taken as independent variables. Multivariate logical regression analysis was carried out. A two-tailed *p*-value of <0.05 was considered statistically significant.

## 3. Results

### 3.1. Participant Characteristics

As shown in Figure 1, there were 16,954 older adults who participated in the 2008 survey; 9778 older adults died, and 10,253 older adults were lost to follow-up. Finally, a total of 2454 older adults completed four waves of follow-up. Among 2454 participants, there were 751 (30.6%) people aged 60 to 69 years old, 1011 (41.2%) people aged 70 to 79 years old, and 692 (28.2%) people over 80 years old. A total of 1147 (46.7%) older adults were male, and 1307 (53.3%) were female. There were 1433 (53.3%) older adults who were married, 77 (3.1%) divorced, 919 (37.4%) widowed, and 25 (1.1%) never married. Most older adults (84.0%) co-reside with their household members. There were 1417 (57.7%) older adults who were satisfied with their lives, and 1393 (56.8%) older adults self-rated as being in good health.

### 3.2. Classification of IC Trajectories

**Figure 2:**
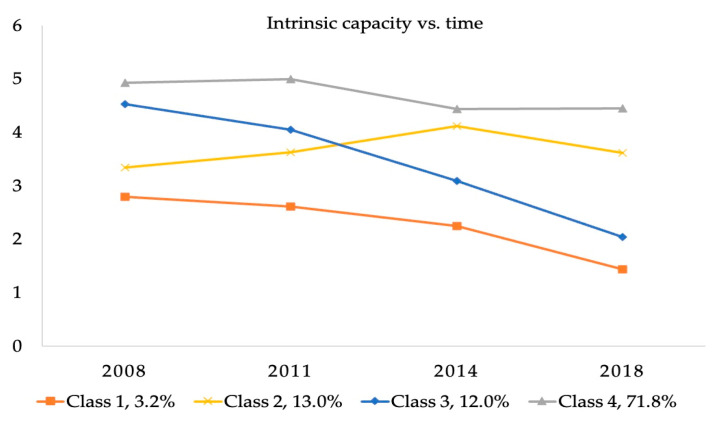
Heterogeneous trajectory groups of IC.

We used the GMM model to classify the IC trajectory of 2454 subjects with possible clustering numbers from 1 to 5 (Table 1). For the GMM model, with the increase in the number of classifications, AIC, BIC, and SABIC decrease gradually, suggesting a better fit of the latter classification. Although a significant *p*-value of VLMR-LRT and BLRT was observed between the 2-class, 3-class, and 4-class, the *p*-value of the 5-class LCGM was greater than 0.05, indicating that a 4-class classification was the most appropriate. Therefore, the GMM with four classes was adopted as the final model (AIC = 29,791.254, BIC = 29,878.336, SABIC = 29,830.677, entropy = 0.729, VLMR-LRT *p* < 0.001, BLRT *p* < 0.001). Therefore, the final four trajectory groups were classified with specific characteristics: class 1, low-level IC (3.2%); class 2, medium-level increasing IC (13.0%); class 3, medium-level decreasing IC (12.0%); and class 4, stable high IC (71.8%) (Figure 2).

### 3.3. Influencing Factors of IC Classification

Univariate analysis of baseline characteristics and IC trajectories showed that there were significant correlations between most factors and IC trajectories (*p* < 0.05), including age, gender, marital status, co-residence with household member(s), financial support, self-rated economic status, smoking, drinking, exercise, social activity, medical service, retirement pension, life satisfaction, and self-rated health (Table 2).

Table 3 presents the results of a multinomial logistic regression model that describes the influencing factors of the IC trajectories. These results revealed significant influencing factors of distinct IC developmental trajectories compared with the low-level IC trajectory. The influencing factor of the medium-level IC trajectory was age (OR = 5.890, 95% CI: 1.344–25.812). The influencing factors of the medium-level decreasing IC trajectory were age (60~69 OR = 11.295, 95% CI: 2.599–49.081; 70~79 OR = 2.087, 95% CI: 1.149–3.791), exercise (OR = 1.944, 95% CI: 1.006–3.756), and social activity (OR = 4.648, 95% CI: 1.072–20.147). The influencing factors of the stable high IC trajectory were age (60~69 OR = 45.947, 95% CI: 10.876–194.110; 70~79 OR = 5.720, 95% CI: 3.275–9.991), gender (OR = 2.576, 95% CI: 1.364–4.865), drinking (OR = 2.630, 95% CI: 1.072–6.450), social activity (OR = 5.905, 95% CI: 1.401–24.888), and self-rated health (OR = 1.860, 95% CI: 1.088–3.180).

## 4. Discussion

In this study, we identified potential trajectory groups of IC and the influencing factors of different trajectory groups. On the whole, four IC trajectories were determined among older adults. Moreover, different influencing factors were found in different IC developmental trajectory groups.

The results of this study showed four IC trajectories: low-level IC, medium-level increasing IC, medium-level decreasing IC, and stable high IC. This finding is consistent with that in the study of Chen et al. [11] involving Taiwanese older adults. Their results identified four classes sharing similar longitudinal IC trajectories: “high-stable” (20.13%), “normal-stable” (40.58%), “sensory dysfunction” (29.53%), and “all dysfunction” (9.76%). However, three IC patterns (“sharp declines in sensory domain”, “sharp declines in locomotion, psychological, cognition, and vitality domains”, and “relatively healthy”) were identified in Yu et al.’s study [31]. Regarding the size of the trajectory groups in our study, the stable high IC group was the largest (71.8%), which was followed by the medium-level increasing IC, medium-level decreasing IC, and low-level IC. The trajectories of the four ICs determined in this study are not consistent with previous studies that identified three classes: low baseline IC with a steeply decreasing trajectory, medium baseline IC with a slightly decreasing trajectory, and high baseline IC with a moderately increasing trajectory [18]. The reason may be that the study population is different; their research subjects were Mexicans aged 50 and above, while ours are Chinese older adults aged 60 and above. Moreover, differences between Chinese and foreign cultural backgrounds and social security systems may lead to differences in the trajectory of IC.

With respect to demographic variables, our study suggested that age was an influencing factor of the medium-level increasing IC trajectory, medium-level decreasing IC trajectory, and stable high IC trajectory compared with the low-level IC trajectory. The association between age and IC was consistent with a previous study, which found that people in early old age were more likely to have better IC [32]. Another study also confirmed this result, which found that the IC of older patients became more impaired with age [33]. It has been suggested that males are significantly associated with a high IC trajectory [5]. Although women have a higher life expectancy than men, women have lower levels of muscle mass, neuroendocrine and hormonal factors than men, and women have higher levels of disability, incidence of comorbidities, higher prevalence of weakness, and more types of drugs, which can have a negative impact on IC.

Lifestyle factors were also significant determinants of IC trajectories in our study. The existing study showed that older adults who drink alcohol are more likely to have high IC. This could be explained by the fact that ethanol initially increases hippocampal acetylcholine release, which could conceivably improve memory performance and cognitive function. Some systematic reviews have confirmed that light to moderate alcohol consumption tends to be protective against cognitive decline and dementia [34,35]. Meanwhile, moderate drinking may be psychologically beneficial because social factors associated with drinking may play a role. Our results showed that social activity is a protective factor in determining the stable high IC trajectory and medium-level decreasing IC trajectory. This finding is consistent with previous studies, which found that older adults with social participation and entertainment activities are more likely to maintain good IC than those without [14]. According to the activity theory, regular activities for older adults are the basis and key to maintaining self-esteem, obtaining psychological satisfaction, and living a long and healthy life, and that maintaining appropriate intelligence and physical strength and participating in certain social activities are the needs of later life, which are not only conducive to their physical health but also conducive to mental health [36]. Moreover, we found that exercise is associated with a medium-level decreasing IC trajectory. In contrast, previous studies reported that exercise is a protective factor for the IC of older adults. The possible explanation is that exercise can improve the physical function, cognitive function, and cardiovascular function in older adults and reduce the incidence of falls, disability, and weakness to maintain a better IC of older adults. A rapid review has shown that physical exercise can be used as an intervention to improve the weakness of older adults and improve the level of IC; that is, walking and endurance interventions have strongly positive effects on IC [37]. However, exercise did not show statistical significance for the high IC trajectory group, perhaps because only 34.7% of older adults regularly exercised in our study.

Older adults with good self-rated health were more likely to be classified as the stable high IC trajectory group. This finding was similar to the results reported in a previous study that self-rated health of older adults is associated with IC and its domains [38]. However, social services and life satisfaction did not determine the IC trajectory groups in our study. The relationships between these factors and IC remain inconclusive in previous studies. Previous studies have shown that social characteristics such as living environment, social relations, security, and social services can have an impact on the IC of older adults [16]. There are several underlying mechanisms for this impact. First, medical services can provide basic medical security for older adults, meet the medical needs of older adults, and maintain their health. Second, retirement pensions are mainly used to ensure the basic livelihood of older adults after retirement and to meet their material needs. Third, social services can ensure the basic health and economic needs of older adults, increase their life satisfaction, alleviate the adverse effects of aging, and achieve better sustainable benefits. Future research should further explore the relationship between social, policy, and other external factors and the IC of older adults.

Although this study represented a step forward in the understanding of IC trajectory patterns considering multinomial heterogeneity, there still existed several limitations. First, because secondary data were used in our study, limited influencing factors of IC were included. Future studies should consider other variables that could impact IC, such as living environment and social support. Second, the influencing factors may change over time, but we only examined the impact of the value of the baseline level on the IC trajectory groups. Future research can further expand cross-cultural comparative studies, long-term follow-up studies, and comprehensive factor analysis to explore the impact of different changes in intrinsic capabilities on outcomes such as morbidity, mortality, and healthcare costs. Third, the calculation method of the composite score of IC failed to consider the weight of each domain. But in fact, the damage of different domains may have different degrees of impact on overall health. Therefore, directly summing the number of normal domains to obtain a composite score may cause a certain deviation in the results. In the future, more research is needed to clarify the impact weights of each domain, and develop a reasonable scoring system to assess IC more objectively and accurately. Finally, in the ICOPE guidelines, weight loss and appetite loss were recommended to measure the vitality domain, while BMI was used in this study. The availability of data limits the consistency with the ICOPE recommendation and may bias the results.

Our research findings, though primarily based on data from older adults in China, hold a certain reference value for populations in other countries. Given China’s unique sociocultural background, including family structure, cultural traditions, lifestyles, and healthcare systems, when applying our results to other populations, it is crucial to note that sociocultural differences may exert some influence on the outcomes. For instance, in some countries, the social status and family roles of older adults may differ from those in China, potentially impacting their IC development trajectories. Nonetheless, the development of IC is a ubiquitous phenomenon, and populations in different countries may undergo similar physiological and psychological changes when facing aging issues. Therefore, our research findings possess certain universal significance in revealing the general patterns of IC development among older adults. In general, this study reveals the developmental trajectories of IC among older adults, enabling policymakers to formulate relevant policy documents that support the maintenance or enhancement of their IC. Meanwhile, given limited medical resources, it is crucial to focus on key target groups with moderate IC, who are more susceptible to changes. Furthermore, by identifying factors influencing different trajectories of IC, this study guides healthcare professionals in developing more targeted and effective care services, such as customized exercise plans, nutrition counseling, and mental health support.

## 5. Conclusions

In conclusion, our findings highlight the dynamic nature of IC by identifying subpopulations of older adults with distinctive changes in IC. Those who have stable high or low IC levels as they enter old age may maintain these levels, so early preventive measures are necessary. In addition, people with intermediate IC levels are more susceptible to change, so they may be a key target group for intervention with limited medical resources. Furthermore, this study confirmed that age, gender, drinking, exercise, social activity, and self-rated health are influencing factors among different IC trajectory groups from a longitudinal perspective. These findings provide a theoretical reference for early evaluation and targeted intervention to improve IC.

## Figures and Tables

**Table 1:** Results of GMM analysis.

Model	AIC	BIC	SABIC	Entropy	VLMR-LRT *p*-Value	BLRT *p*-Value	Classification Probability
1-class	31,036.680	31,071.513	31,052.449	/	/	/	1
2-class	30,003.871	30,056.120	30,027.525	0.757	<0.001	<0.001	0.195/0.805
3-class	29,904.699	29,974.365	29,936.238	0.749	0.0273	<0.001	0.742/0.027/0.231
4-class	29,791.254	29,878.336	29,830.677	0.729	<0.001	<0.001	0.032/0.130/0.120/0.718
5-class	29,781.631	29,886.129	29,828.939	0.662	0.055	<0.001	0.037/0.174/0.639/0.123/0.027

**Table 2:** Results of univariate analysis of baseline features and IC trajectories.

	Group	All (n)	Group 1 (n, %)	Group 2 (n, %)	Group 3 (n, %)	Group 4 (n, %)	χ^2^	*p*-Value
Sociodemographic characteristics								
Age	60~69	751 (30.6)	2 (0.3)	39 (5.2)	57 (7.6)	653 (86.9)	323.277	<0.001
	70~79	1011 (41.2)	20 (1.9)	106 (10.5)	106 (10.5)	779 (77.1)		
	≥80	692 (28.2)	57 (8.2)	173 (25)	132 (19.1)	330 (47.7)		
Gender	Male	1147 (46.7)	20 (1.7)	79 (6.9)	84 (7.3)	964 (84.0)	160.320	<0.001
	Female	1307 (53.3)	59 (4.5)	239 (18.3)	211 (16.1)	798 (61.1)		
Marital status	Married	1433 (58.4)	26 (1.8)	127 (8.9)	124 (8.7)	1156 (80.7)	165.032	<0.001
	Divorced	77 (3.1)	1 (1.3)	3 (3.9)	9 (11.7)	64 (83.1)		
	Widowed	919 (37.4)	51 (5.5)	185 (20.1)	159 (17.3)	524 (57.0)		
	Never married	25 (1.1)	1 (4)	3 (12)	3 (12)	18 (72)		
Co-residence with household member(s)	Yes	2061 (84.0)	66 (3.2)	245 (11.9)	225 (10.9)	1525 (74.0)	33.068	<0.001
	No	393 (16.0)	13 (3.3)	73 (18.6)	70 (17.8)	237 (60.3)		
Financial support	Yes	1915 (78.0)	53 (2.8)	243 (12.7)	213 (11.1)	1406 (73.4)	15.050	0.002
	No	539 (22.0)	26 (4.8)	75 (13.9)	82 (15.2)	356 (66.0)		
Self-rated economic status	Rich	319 (13.0)	14 (4.4)	38 (11.9)	28 (8.8)	239 (74.9)	40.980	<0.001
	Not bad	1742 (71.0)	44 (2.5)	222 (12.7)	191 (11.0)	1285 (73.8)		
	Poor	393 (16.0)	21 (5.3)	58 (14.8)	76 (19.3)	238 (60.6)		
Lifestyles								
Smoking	Yes	554 (22.6)	11 (2.0)	35 (6.3)	54 (9.7)	454 (82.0)	41.075	<0.001
	No	1900 (77.4)	68 (3.6)	283 (14.9)	241 (12.7)	1308 (68.8)		
Drinking	Yes	542 (22.1)	6 (1.1)	44 (8.1)	41 (7.6)	451 (83.2)	46.323	<0.001
	No	1912 (77.9)	73 (3.8)	274 (14.3)	254 (13.3)	1311 (68.6)		
Exercise	Yes	852 (34.7)	14 (1.6)	107 (12.6)	96 (11.3)	635 (74.5)	12.202	0.007
	No	1602 (65.3)	65 (4.1)	211 (13.2)	199 (12.4)	1127 (70.3)		
Social activity	Yes	461 (18.8)	2 (0.4)	39 (8.5)	39 (8.5)	381 (82.6)	37.832	<0.001
	No	1993 (81.2)	77 (3.9)	279 (14.0)	256 (12.8)	1381 (69.3)		
Social services								
Medical service	Yes	2291 (93.4)	66 (2.9)	290 (12.7)	260 (11.3)	1675 (73.1)	35.898	<0.001
	No	163 (6.6)	13 (8.0)	28 (17.1)	35 (21.5)	87 (53.4)		
Retirement pension	Yes	417 (17.0)	4 (1.0)	48 (11.5)	27 (6.5)	338 (81.1)	27.630	<0.001
	No	2037 (83.0)	75 (3.7)	270 (13.3)	268 (13.2)	1424 (69.9)		
Life satisfaction	Good	1417 (57.7)	47 (3.3)	176 (12.4)	146 (10.3)	1048 (74.0)	11.253	0.010
	Bad	1037 (42.3)	32 (3.1)	142 (13.7)	149 (14.4)	714 (68.8)		
Self-rated health	Good	1393 (56.8)	37 (2.7)	161 (11.6)	131 (9.4)	1064 (76.3)	35.823	<0.001
	Bad	1061 (43.2)	42 (4.0)	157 (14.8)	164 (15.5)	698 (65.8)		

**Table 3:** Multinomial logistic regression results.

Measures	Class 1 OR (95% CI)	Class 2 OR (95% CI)	Class 3 OR (95% CI)	Class 4 OR (95% CI)
Sociodemographic characteristics				
Age (≥80)				
60~69	ref	5.890 (1.344–25.812) *	11.295 (2.599–49.081) **	45.947 (10.876–194.110) ***
70~79	ref	1.580 (0.874–2.854)	2.087 (1.149–3.791) *	5.720 (3.275–9.991) ***
Gender (female)				
Male	ref	0.834 (0.422–1.648)	0.920 (0.464–1.826)	2.576 (1.364–4.865) **
Marital status (never married)				
Married	ref	2.230 (0.194–25.685)	3.131 (0.269–36.498)	5.203 (0.548–49.379)
Divorced	ref	1.236 (0.046–33.514)	5.208 (0.216–125.534)	7.110 (0.358–141.191)
Widowed	ref	1.797 (0.158–20.422)	2.555 (0.222–29.398)	3.128 (0.333–29.353)
Co-residence with household member(s) (no)				
Yes	ref	0.536 (0.262–1.096)	0.514 (0.250–1.057)	0.577 (0.289–1.149)
financial support (no)				
Yes	ref	1.474 (0.741–2.933)	1.681 (0.843–3.352)	1.382 (0.723–2.639)
Self-rated economic status (poor)				
Not bad	ref	0.566 (0.207–1.493)	0.376 (0.138–1.024)	0.633 (0.251–1.593)
Rich	ref	1.251 (0.597–2.619)	0.869 (0.418–1.808)	1.484 (0.739–2.977)
Lifestyles				
Smoking (no)				
Yes	ref	0.570 (0.248–1.307)	1.010 (0.449–2.273)	0.710 (0.331–1.522)
Drinking (no)				
Yes	ref	2.306 (0.902–5.898)	1.866 (0.725–4.804)	2.630 (1.072–6.450) *
Exercise (no)				
Yes	ref	1.872 (0.973–3.602)	1.944 (1.006–3.756) *	1.679 (0.896–3.145)
Social activity (no)				
Yes	ref	3.807 (0.878–16.498)	4.648 (1.072–20.147) *	5.905 (1.401–24.888) *
Social services				
Medical service (no)				
Yes	ref	1.590 (0.679–3.722)	1.370 (−0.594–3.163)	2.061 (0.924–4.598)
Retirement pension (no)				
Yes	ref	0.427 (0.139–1.313)	0.809 (0.255–2.567)	0.505 (0.169–1.504)
Life satisfaction (bad)				
Good	ref	0.663 (0.361–1.217)	0.652 (0.354–1.201)	0.753 (0.423–1.342)
Self-rated health (bad)				
Good	ref	1.277 (0.726–2.248)	1.072 (0.606–1.898)	1.860 (1.088–3.180) *

Note: OR = odds ratio; CI = confidence interval; * *p* < 0.05, ** *p* < 0.01, *** *p* < 0.001.

## Data Availability

The data that support the findings of this study are openly available at https://doi.org/10.18170/DVN/WBO7LK (accessed on 1 March 2024).
